# Combined resection of the transpancreatic common hepatic artery preserving the gastric arterial arcade without arterial reconstruction in hepatopancreatoduodenectomy: a case report

**DOI:** 10.1186/s40792-018-0474-8

**Published:** 2018-06-26

**Authors:** Takashi Miyata, Yusuke Yamamoto, Teiichi Sugiura, Yukiyasu Okamura, Takaaki Ito, Ryo Ashida, Sunao Uemura, Yoshiyasu Kato, Katsuhisa Ohgi, Atsushi Kohga, Tsuneyuki Uchida, Shusei Sano, Katsuhiko Uesaka

**Affiliations:** Division of Hepato-Biliary-Pancreatic Surgery, Shizuoka Cancer Center, 1007, Shimo-Nagakubo, Sunto-Nagaizumi, Shizuoka, 4118777 Japan

**Keywords:** Transpancreatic common hepatic artery, Hepatomesenteric trunk, Gastric arterial arcade, Hepatopancreatoduodenectomy, Neuroendocrine tumor

## Abstract

**Background:**

Surgeons sometimes must plan pancreatoduodenectomy (PD) for patients with a variant common hepatic artery (CHA) branching from the superior mesenteric artery (SMA) penetrating the pancreatic parenchyma, known as a transpancreatic CHA (tp-CHA).

**Case presentation:**

A 67-year-old man was admitted to our hospital because of liver dysfunction. A duodenal tumor was identified by gastrointestinal endoscopy, and a biopsy revealed a neuroendocrine tumor. Computed tomography showed multiple metastases in the left three sections of the liver. As an anatomical variant, the CHA branched from the SMA and passed through the parenchyma of the pancreatic head, and all hepatic arteries branched from the CHA. Furthermore, the arcade between the left and right gastric artery (RGA) was detected, and the RGA branched from the root of the left hepatic artery. PD and left trisectionectomy of the liver were performed. The tp-CHA was resected with the pancreatic head, and the gastric arterial arcade was preserved to maintain the right posterior hepatic arterial flow. Postoperatively, there were no signs of hepatic ischemia.

**Conclusions:**

When planning PD, including hepatopancreatoduodenectomy, for patients with a tp-CHA, surgeons should simulate various situations for maintaining the hepatic arterial flow. The preservation of the gastric arterial arcade is an option for maintaining the hepatic arterial flow to avoid arterial reconstruction.

**Electronic supplementary material:**

The online version of this article (10.1186/s40792-018-0474-8) contains supplementary material, which is available to authorized users.

## Background

The common trunk formed by the common hepatic artery (CHA) and the superior mesenteric artery (SMA) is referred to as the hepatomesenteric trunk; this is only found in 1.5–2.3% of the population [1, 2]. Among such individuals, only a few patients have the CHA passing fully through the pancreatic parenchyma (transpancreatic CHA [tp-CHA]) [1, 3]. During pancreatoduodenectomy (PD) (including hepatopancreatoduodenectomy [HPD]) for patients with tp-CHA, it is necessary to consider the surgical procedure to maintain the hepatic arterial flow, including the preservation of the CHA separating from pancreatic parenchyma [4], reconstruction of the hepatic artery after combined resection of tp-CHA [4, 5], and the preservation of the collateral circulation after combined resection of tp-CHA [2], in order to avoid hepatic ischemia and lethal complications [4, 6].

We herein report a case of duodenal neuroendocrine tumor (NET) with multiple liver metastases for a patient with a tp-CHA. The patient was successfully treated with PD and left trisectionectomy with caudate lobectomy combined resection of the tp-CHA and preservation of the gastric arterial arcade in order to maintain the hepatic arterial flow.

## Case presentation

**Fig. 1:**
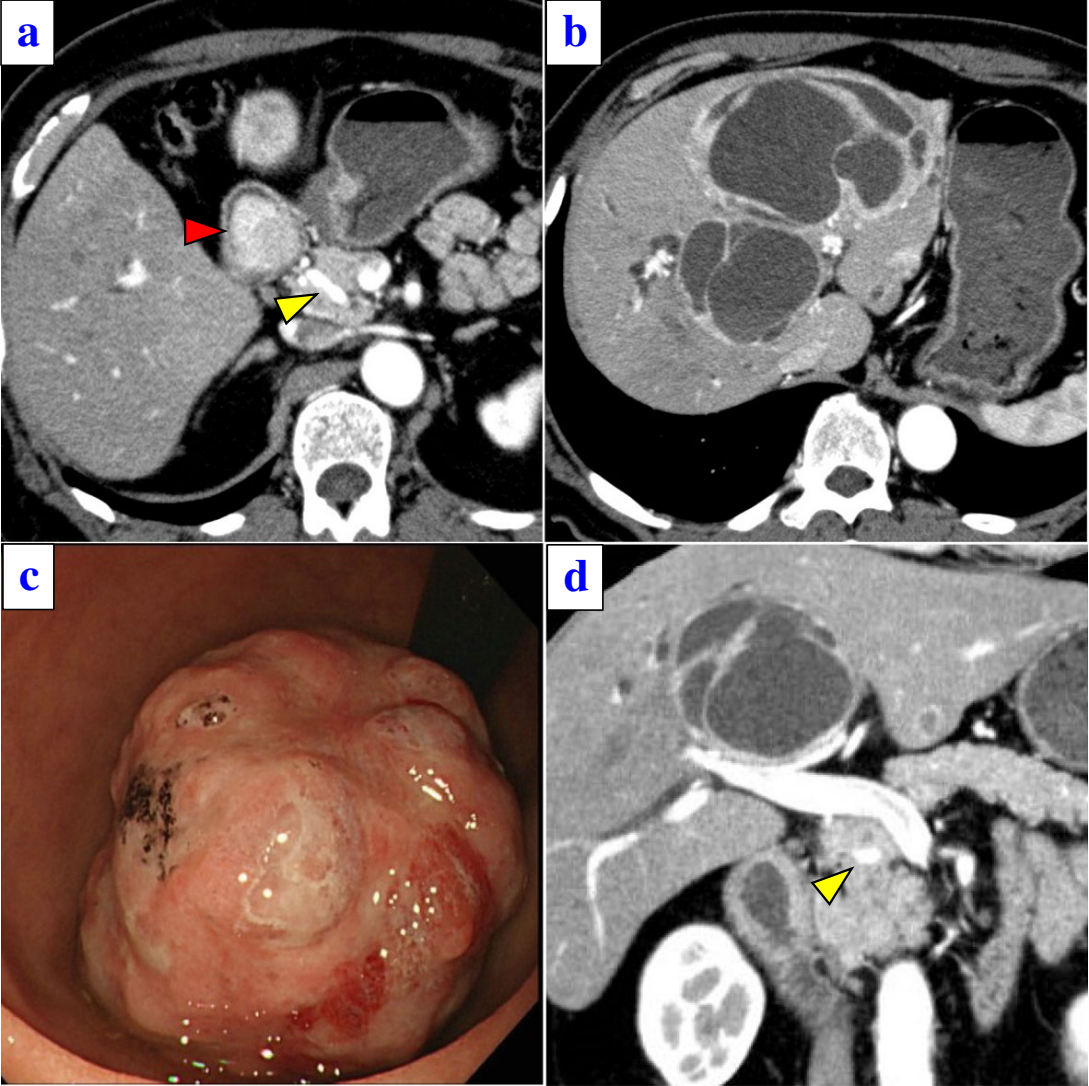
**a** A duodenal tumor with increased contrast enhancement was observed (red arrow). In addition, the common hepatic artery (CHA) (yellow arrow) branched from the superior mesenteric artery (SMA), passing through the pancreatic head. **b** Abdominal CT showed multiple metastases in the left three lobes of the liver. **c** A 3.5-cm-diameter type 1 tumor in the descending portion of the duodenum was revealed by gastroscopy. **d** A coronal image. The yellow arrow is the CHA branching from the SMA, passing through the pancreatic head

**Fig. 2:**
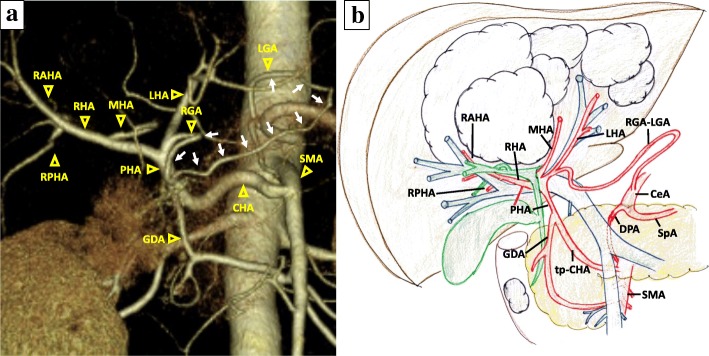
**a** A preoperative three-dimensional CT angiogram. The gastric arterial arcade is shown (white arrows). **b** A preoperative schematic illustration. LGA left gastric artery, RGA right gastric artery, GDA gastroduodenal artery, PHA proper hepatic artery, LHA left hepatic artery, MHA middle hepatic artery, RHA right hepatic artery, RAHA right anterior hepatic artery, RPHA right posterior hepatic artery, CeA celiac artery, SpA splenic artery, DPA dorsal pancreatic artery, tp-CHA transpancreatic CHA

A 67-year-old man was admitted to our hospital because of liver dysfunction during a screening examination. Enhanced abdominal computed tomography (CT) revealed a hypervascular mass of 35 mm in diameter in the descending portion of the duodenum (Fig. 1a), and the left three sections of the liver were occupied by multiple cystic tumors with contrast enhancement of the cystic wall, 13 cm in diameter (Fig. 1b). A duodenal tumor was identified on gastrointestinal endoscopy (Fig. 1c), and a biopsy revealed a NET. The serum levels of insulin, gastrin, and glucagon were within normal ranges. CT did not initially reveal evidence of pancreatic invasion between the tumor and the pancreas; however, irregularities of the duodenal wall and swelling of the lymph nodes around the pancreatic parenchyma were observed. Thus, the patient was diagnosed with non-functional duodenal NET with multiple liver metastases, T2N1M1 stage IV (UICC 8th). In addition, CT revealed the anatomical variation of the CHA, which branched from the SMA and ran fully through the head of the pancreatic parenchyma (Fig. 1a, d, Additional file 1 Figure S1). The CHA branches into the left hepatic artery (LHA), the middle hepatic artery (MHA), and the right hepatic artery (RHA) (Fig. 2a, b). Furthermore, a developed gastric arterial arcade, 4 mm in diameter, was found between the left gastric artery (LGA) and the right gastric artery (RGA). The RGA was branched from a distal portion at a distance of 10 mm from the root of the LHA (Fig. 2a, b). Incidentally, we did not observe stenosis of the celiac axis due to compression by the median arcuate ligament. We planned PD and left trisectionectomy with caudate lobectomy combined resection of the tp-CHA with the preservation of the gastric arterial arcade in order to maintain arterial flow of the remnant liver, preserving the route of the celiac artery to the right posterior hepatic artery (RPHA) via the gastric arterial arcade from the LGA to the RGA, LHA, and RHA. If the hepatic arterial flow could not be maintained by this route, the preservation of the tp-CHA by separating from pancreatic parenchyma or arterial reconstruction using radial artery graft between CHA and RHA was planned. Four weeks after percutaneous transhepatic portal embolization, surgery was carried out.

**Fig. 3:**
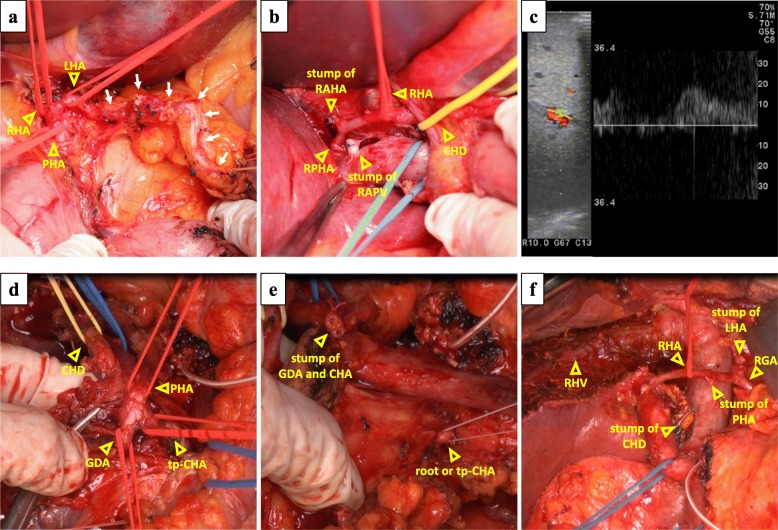
**a** The gastric arterial arcade (white arrows) was exposed, and the LHA, RHA, and PHA were taped. **b** The RPHA was supraportal, and the RAHA, RAPV, and right anterior portal vein were divided. CHD common hepatic duct. **c** The hepatic arterial signal under PHA clamping was confirmed. **d** The specimen was left connected by only the PHA, CHA, and CHD. **e** We divided the PHA, GDA, and root of tp-CHA. **f** An image was obtained after the specimen was removed. RHV right hepatic vein

After laparotomy, the gastric arterial arcade was exposed and encircled, and the LHA, RHA, and proper hepatic artery (PHA) were encircled (Fig. 3a). The LHA was divided at the distal side of the origin of the RGA. The MHA and the right anterior hepatic artery (RAHA) were also divided. The left portal branch and the right anterior portal branch were divided (Fig. 3b). The liver was transected, and the left hepatic duct and right anterior hepatic duct were divided. The left trisections and caudate lobe were anatomically resected. After clamping the PHA, the hepatic arterial signals of the RPHA via the gastric arterial arcade were confirmed by intraoperative Doppler ultrasonography (Fig. 3c). After trisectionectomy and caudate lobectomy, PD was performed. The pancreatic head was dissected from the SMA after the upper jejunum was divided. The pancreas was divided in front of the SMV. Finally, the specimen was only connected by the tp-CHA and the common hepatic duct (CHD) (Fig. 3d). The hepatic arterial signals of the RPHA was maintained after clamping the PHA. The PHA and the origin of CHA were divided, and the tp-CHA was taken out with the pancreatic head (Fig. 3e). The CHD was divided, and the specimen was removed (Fig. 3f). Reconstruction was performed via modified Child’s method. The operative time was 1072 min and the intraoperative blood loss was 3052 ml, and red blood cell transfusion was performed (1680 ml).

**Fig. 4:**
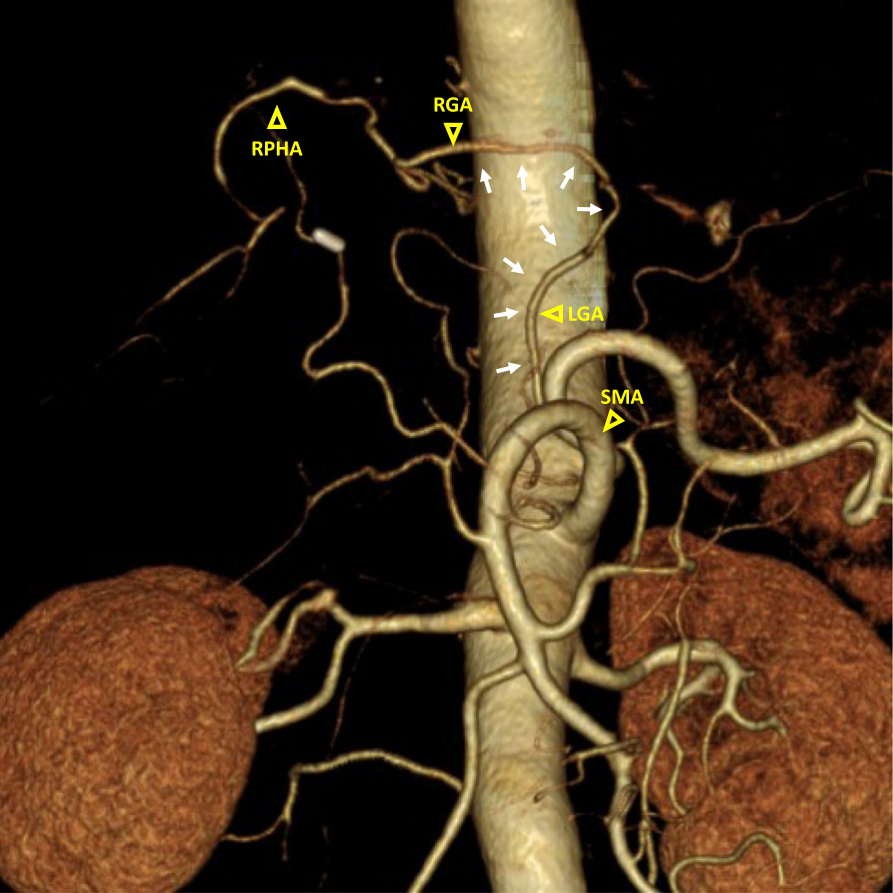
A postoperative three-dimensional CT angiogram. The gastric arterial arcade is shown (white arrows)

Postoperatively, the patient developed pancreatic fistula (Clavien-Dindo IIIa) and biliary leak (Clavien-Dindo IIIa), and these complications were treated conservatively. There were no signs of hepatic ischemia. The patient was discharged on postoperative day 39. The pathological diagnosis was duodenal neuroendocrine tumor G2 with multiple liver metastases. The Ki-67 labeling index was < 20%, and staining for chromogranin A and synaptophysin were positive. There was no evidence of invasion of the pancreatic parenchyma; however, the duodenal tumor was confined to the MP layer, and one of the 25 examined lymph nodes was positive, and moderate lymphovascular invasion was observed. The final diagnosis was pMP, med, INFa, ly1, v2, pPM0, pDM0, and pEM0. The patient has shown no recurrence in the 22 months since the operation. Enhanced abdominal CT at 4 months after surgery revealed the blood flow of the RPHA via the gastric arcade (Fig. 4).

### Discussion

Over the years, several authors have described variations in the hepatic arterial anatomy; a CHA arising from the SMA—called the hepatomesenteric type—is a rare clinical entity. Yang et al. and Hiatt et al. reported that this condition was observed in only 31 of 1324 patients and 15 of 1000 patients, respectively [1, 2]. A CHA passing through the pancreatic head parenchyma, tp-CHA, is even rarer; Yang et al. [1] reported that among 31 patients with the hepatomesenteric type, only 3 had this condition.

When PD is scheduled in such patients with tp-CHA, it is important to maintain the arterial supply to the liver. Surgeons should preoperatively determine whether to preserve or perform combined resection of the tp-CHA. Tp-CHA preservation was selected in several previous reports [4, 5, 7]. This surgical procedure is technically feasible; however, there is a risk of a positive surgical margin or insufficient lymph node dissection and a tendency for increased intraoperative blood loss during the separation of the pancreatic parenchyma. If the tp-CHA is resected, reconstruction is usually necessary in order to maintain the hepatic arterial flow. Previous reports [5, 8, 9] have described successful arterial reconstruction after CHA resection during PD; however, such procedures are associated with an increased risk of thromboembolism, which can lead to a fatal outcome, especially in HPD [7]. In contrast, when collateral circulation develops, surgeons can perform combined resection of the tp-CHA, preserving the collateral circulation without arterial reconstruction. Several reports have recommended preoperative embolization of CHA in order to maintain the hepatic arterial flow through enlarged collateral arteries [10]. Although preoperative embolization can increase the liver arterial flow through collateral arteries, it is not routinely recommended because of the risk of complications, which includes the migration of embolic material [11, 12].

A developed gastric arcade or pancreaticoduodenal arcade is frequently seen in patients with the stenosis of the CHA due to factors such as compression by the median arcuate ligament [13]. There are only a few cases in which the hepatomesenteric trunk and the tp-CHA and the association between the tp-CHA and the development of a gastric arterial arcade have not been reported. On the other hand, Miyamoto et al. reported the case of a patient with pancreatic head cancer with a CHA arising from the SMA who underwent radical PD combined with the resection of the CHA, in which the hepatic arterial flow was maintained via the gastric arterial arcade [14]. In this report, the patient did not have a developed gastric arterial arcade; however, the hepatic arterial flow via the gastric arterial arcade was sufficient and hepatic ischemia was not detected after the operation. Considering this case, even if the patients with tp-CHA do not have a developed gastric arterial arcade, surgeons may be able to preserve hepatic arterial flow via the gastric arterial arcade alone. If the hepatic arterial flow via the gastric arterial arcade alone is adequate after clamping the PHA, the combined resection of the tp-CHA can be considered, even if the gastric arcade is not developed before surgery. In cases in which the hepatic arterial flow is not adequate, the preservation of the tp-CHA or arterial reconstruction should be considered.

When performing HPD, a PD-first procedure before hepatectomy is generally performed, as this approach is anatomically rational [15]. However, in the present case, performing hepatectomy after PD carried a risk of the arterial supply to the liver being reduced during hepatectomy. Had we chosen a PD-first procedure and the hepatic arterial flow not been maintained after CHA resection, it would have been necessary to perform arterial reconstruction before liver transection. This method is associated with a risk of injury to the reconstructed artery and thrombosis during liver transection. Given the above, we opted to perform hepatectomy before PD in our patient with a tp-CHA undergoing HPD.

In the procedure for separating the tp-CHA from the pancreatic parenchyma entirely, the surgeon should be concerned about the increasing rate of hemorrhage, surgery time, and the risk of injury to the tp-CHA. The surgical reconstruction of the hepatic artery when performing HPD is also associated with a high degree of risk. The association between tp-CHA and gastric arterial arcade was recognized on preoperative CT scans; the development of this collateral circulation may have the potential to prevent ischemia-related liver complications. From these points of view, the preoperative identification of the developed arcade of the gastric arteries helps in planning an appropriate operative procedure, and this procedure seems to be a viable and simple option. To our knowledge, this is the first report of PD combined with resection of a tp-CHA without preoperative embolization. Furthermore, this is also the first report of HPD for a patient with a tp-CHA. The preoperative identification of the developed arcade of the gastric arteries helps in planning the appropriate operative procedure when PD is scheduled for patients with a tp-CHA.

## Conclusions

When planning PD for patients with a tp-CHA, a precise preoperative evaluation and the adoption of the surgical strategy and technique for each individual case are critical to preserving the hepatic arterial flow.

## Additional file

Additional file 1: A preoperative abdominal contrast-enhanced CT presenting the CHA branched from the SMA and passed through the parenchyma of pancreatic head. (PDF 1833 kb)
