# Supplementary figures and images for: Combined resection of the transpancreatic common hepatic artery preserving the gastric arterial arcade without arterial reconstruction in hepatopancreatoduodenectomy: a case report
Source: Surg Case Rep. 2018 Jun 26;4:64. doi: 10.1186/s40792-018-0474-8 (PMC6020092; doi:10.1186/s40792-018-0474-8)

Figure S1

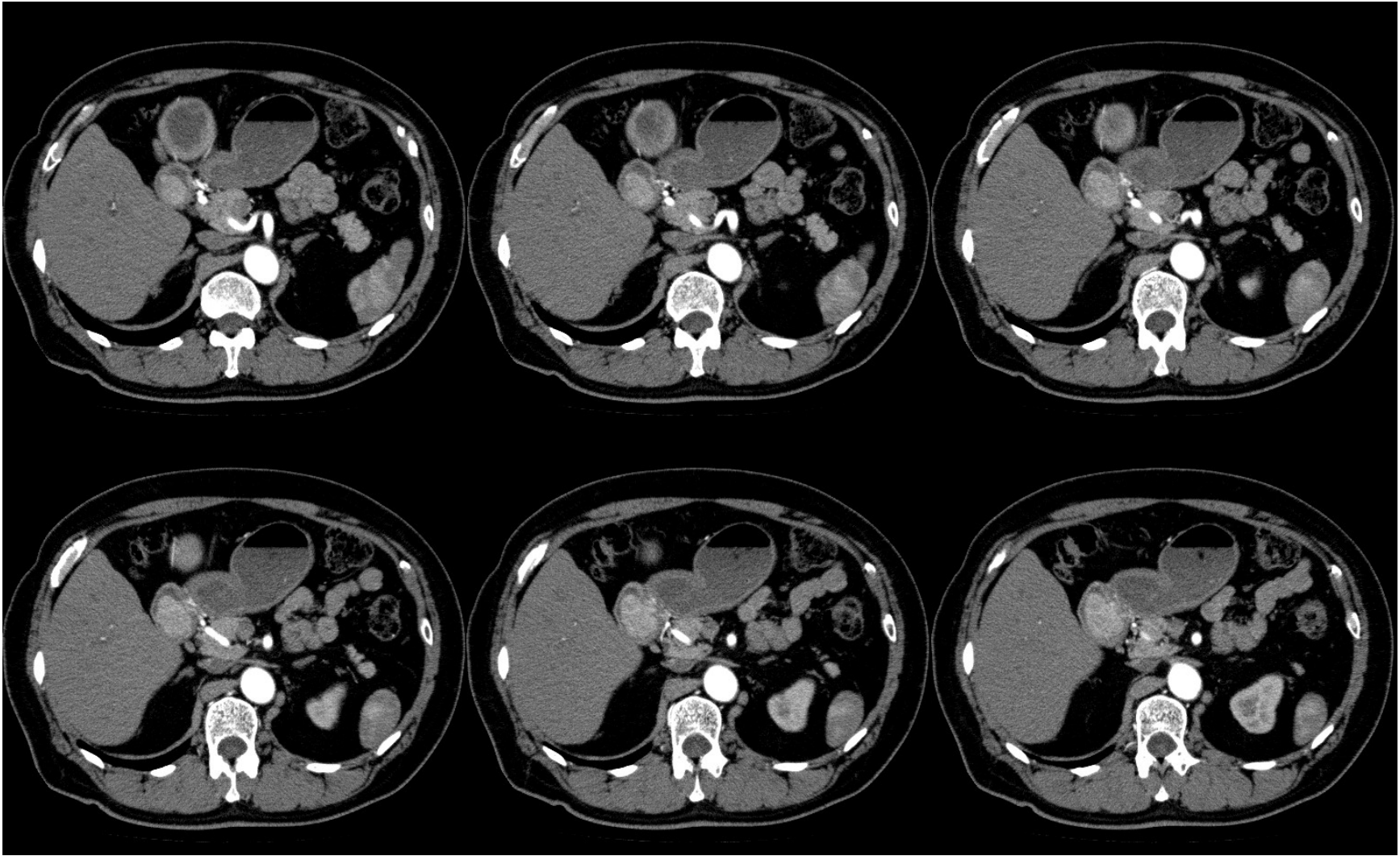

Supplement: Supplementary file 1 — A preoperative abdominal contrast-enhanced CT presenting the CHA branched from the SMA and passed through the parenchyma of pancreatic head. (PDF 1833 kb) [file 40792_2018_474_MOESM1_ESM.pdf]
